# What the papers say

**DOI:** 10.1093/jhps/hnx006

**Published:** 2017-03-27

**Authors:** Ajay Malviya

The Journal of Hip Preservation Surgery (JHPS) is not the only place where work in the field of hip preservation may be published. Although our aim is to offer the best of the best, we continue to be fascinated by work that finds its way into journals other than our own. There is much to learn from it so *JHPS* has selected six recent and topical articles for those who seek a brief summary of what is taking place in our ever-fascinating world of hip preservation. What you see here are the mildly edited abstracts of the original articles, to give them what *JHPS* hopes is a more readable feel. If you are pushed for time, what follows should take you no more than 10 min to read. So here goes …

## DOES PLATELET RICH PLASMA (PRP) WORK IN GLUTEUS MEDIUS TENDINOPATHY?

Lee et al. [1] from the Hospital for Special Surgery, New York prospectively assessed the efficacy of ultrasound-guided intratendinous PRP injections as treatment for chronic recalcitrant gluteus medius tendinopathy with/without partial tears of the tendon. Patients had moderate to severe lateral hip pain for longer than 3 months. All participants were assessed pre- and post-injection with four outcome measures: modified Harris Hip Score (mHHS), Hip Outcome Score-Activities of Daily Living subscale (HOS-ADL), Hip Outcome Score-Sport-Specific subscale (HOS-Sport), and the International Hip Outcome Tool-33 (iHOT-33). Demographic data, including age, sex, height, weight, body mass index, and smoking status, were also collected.

A total of 21 patients were included in the study, with a mean follow-up of 19.7 months (range, 12.1–32.3 months). The mean improvements from preinjection to post-injection follow-up were 56.7–74.1 for mHHS, 68.9–84.1 for HOS ADL, 45.5–66.7 for HOS-Sport, and 34.06–66.33 for iHOT-33. All mean outcome measure improvements were clinically and statistically significant (*P* < 0.001). Length of follow-up was positively correlated with improvements in HOS-ADL (*P* = 0.021) and HOS-Sport (*P* = 0.004) scores. No adverse events were observed during or after the procedure.

The authors concluded in this registry study with prospective follow-up that ultrasound-guided intratendinous PRP injections were a safe and effective treatment option for chronic recalcitrant gluteus medius tendinopathy due to moderate to severe tendinosis and/or partial tendon tears. However, they did feel the need for well-powered randomized controlled studies to confirm their findings and further define the ideal candidates for this treatment.

## REGENERATIVE THERAPY FOR ACETABULAR CHONDRAL DEFECT ASSOCIATED WITH FEMOROACETABULAR IMPINGEMENT

Fontana from Italy [2] has looked at the role of Autologous Membrane Induced Chondrogenesis (AMIC) for the treatment of acetabular chondral defects found during arthroscopy for femoroacetabular impingement. Between 2008 and 2014, 201 patients out of 583 have been arthroscopically treated with the AMIC procedure for grade III and/or IV acetabular chondral lesions. Patient age was between 18 and 50 years; acetabular chondral lesion size was between 2 and 4 cm^2^; radiological Tonnis degree of osteoarthritis was ≤ 2.

The mean follow-up of the entire group of 201 patients was 5 years (from 2 to 8). Significant improvement, as measured by the mHHS, was observed at 6 months in comparison to preoperative levels (80.3) (*P* < 0.001). Continuous improvement with respect to each previous evaluation time point was seen, reaching the highest improvement level at the 3-year follow-up (85.5). The mean mHHS improvement recorded at the 5-year follow-up compared with preoperative scores was 39.1.

The authors concluded that AMIC is a valid procedure to repair medium-sized chondral defects on the acetabular side of the hip found during treatment of FAI and lead to long-term favourable outcomes.

## PERIOPERATIVE PAIN RELIEF AFTER HIP ARTHROSCOPY

Two prospective randomized controlled trials from different parts of the world have looked at different modalities for improving pain after hip arthroscopy.

Researchers from Tel Aviv Sourasky Medical Center, Israel [3] have examined the efficacy of intra-articular and periacetabular blocks for postoperative pain control after hip arthroscopy.

Forty-two consecutive patients scheduled for hip arthroscopy were randomized into two postoperative pain control groups. One group received preemptive intra-articular 20 ml of bupivacaine 0.5% injection, and the second group received preemptive periacetabular 20 ml of bupivacaine 0.5% injection. Before closure, all patients received an additional dose of 20 ml of bupivacaine 0.5% intra articularly. Data were compared with respect to postoperative pain with visual analog scale (VAS) and analgesic consumption, documented in a pain diary for 2 weeks after surgery.

Twenty-one patients were treated with intra-articular injection, and 21 with peri-acetabular injection. There were no significant differences with regards to patient demographics or surgical procedures. VAS scores recorded during the first 30-min postoperatively and 18 h after surgery were significantly lower in the periacetabular group compared with in the intra-articular group (0.667 versus 2.11; *P* = 0.045 and 2.6 versus 4.79; *P* < 0.009). There were no differences between the groups with regard to analgesic consumption.

The authors concluded that periacetabular injection of bupivacaine 0.5% was superior to intra-articular injection in pain reduction after hip arthroscopy at 30 min and 18 h, postoperatively. However, total analgesic consumption over the first two postoperative weeks and VAS pain measurements were not significantly affected.

In contrast, research carried out in Addenbrooke’s Hospital, Cambridge [4] has compared the efficacy of fascia iliaca compartment block (FICB) versus local anaesthetic infiltration (LAI) after hip arthroscopic surgery. Participants were randomized to receiving either FICB or LAI of the portal tracts with local anesthetic. Supplemental analgesia was also used in both groups on an on-demand basis. The primary outcome measure was the postoperative level of pain as assessed by numeric pain score at 1, 3, 6, and 24 h after the procedure in both groups. Secondary outcome measures were the frequency and the dose of morphine and other medications consumed at 1 and 24 h after surgery as well as any other adverse events relating to pain or medications used for pain relief in both the groups.

Interestingly, the study had to be terminated early because there was a significant statistical difference in the primary outcome measure after the recruitment of 46 patients: 20 in the LAI group and 26 in the FICB group. Severity of pain in the FICB group was higher especially during the first hour postoperatively (*P* = 0.02). This was associated with a higher consumption of opioids and other analgesics, which resulted in more side effects such as nausea and vomiting.

The authors concluded that LAI provided a better analgesia after arthroscopic surgery of the hip in comparison with FICB and was also associated with reduced consumption of opioids and a lower rate of side effects.

## SYSTEMATIC REVIEW OF PAIN, ACTIVITIES OF DAILY LIVING AND SPORT FUNCTION AT DIFFERENT TIME POINTS AFTER HIP ARTHROSCOPY FOR FEMOROACETABULAR IMPINGEMENT

In a collaborative work from Denmark and Switzerland [5], researchers aimed to investigate pain, activities of daily living (ADL) function, sport function, quality of life and satisfaction at different time points after hip arthroscopy in patients with femoroacetabular impingement (FAI).

Systematic review with meta-analysis was conducted and weighted mean differences between preoperative and postoperative outcomes were calculated and used for meta-analysis.

Twenty-six studies (22 case series, 3 cohort studies, 1 randomized controlled trial (RCT)) were included in the systematic review and 19 in the meta-analysis. Clinically, relevant pain and ADL function improvements were first reported between 3 and 6 months, and sport function improvements between 6 months and 1 year after surgery. It is not clear when quality of life improvements were first achieved. On an average, residual mild pain and ADL and sport function scores lower than their healthy counterparts were reported by patients following surgery. Postoperative patient satisfaction ranged from 68% to 100%.

The authors concluded that on an average, patients reported earlier pain and ADL function improvements, and slower sport function improvements after hip arthroscopy for FAI. However, average scores from patients indicate residual mild hip pain and/or hip function lower than their healthy counterparts after surgery. The authors recommend that owing to the current low level of evidence, future RCTs and cohort studies should investigate the effectiveness of hip arthroscopy in patients with FAI.

## PATIENT-REPORTED OUTCOMES OF PERIACETABULAR OSTEOTOMY FROM THE PROSPECTIVE ANCHOR COHORT STUDY

Current literature describing periacetabular osteotomy (PAO) is mostly limited to retrospective case series [6]. Larger, prospective cohort studies are needed to provide better clinical evidence regarding this procedure. The goals of the current study were to (1) report minimum 2-year patient-reported outcomes (pain, hip function, activity, overall health, and quality of life), (2) investigate preoperative clinical and disease characteristics as predictors of clinical outcomes, and (3) report the rate of early failures and reoperations in patients undergoing contemporary PAO surgery.

A large, prospective, multicenter cohort of PAO procedures was established, and outcomes at a minimum of 2 years were analysed. A total of 391 hips were included for analysis (79% of the patients were female, and the average patient age was 25.4 years). Patient-reported outcomes, conversion to total hip replacement, reoperations, and major complications were documented. Variables with a *P* values of ≤ 0.10 in the univariate linear regressions were included in the multivariate linear regression. The backward stepwise selection method was used to determine the final risk factors of clinical outcomes.

Clinical outcome analysis demonstrated major clinically important improvements in pain, function, quality of life, overall health, and activity level. Increasing age and a body mass index status of overweight or obese were predictive of improved results for certain outcome metrics. Male sex and mild acetabular dysplasia were predictive of lesser improvements in certain outcome measures. Three (0.8%) of the hips underwent early conversion to total hip arthroplasty, 12 (3%) required reoperation, and 26 (7%) experienced a major complication.

This large, prospective cohort study has demonstrated the clinical success of contemporary PAO surgery for the treatment of symptomatic acetabular dysplasia. Patient and disease characteristics demonstrated predictive value that should be considered in surgical decision-making.

## APPLICATION OF A 3-DIMENSIONAL PRINTED NAVIGATION TEMPLATE IN BERNESE PERIACETABULAR OSTEOTOMIES: A CADAVERIC STUDY

Zhou et al. [7] from Kunming, China aimed to describe the application of 3D printed templates for intraoperative navigation and simulation of periacetabular osteotomies (PAOs) in a cadaveric model. Five cadaveric specimens (10 sides) underwent thin-slice computed tomographic scans of the ala of ilium downwards to the proximal end of femoral shaft. Bernese PAO was performed. Using Mimics v10.1 software (Materialise, Leuven, Belgium), 3D computed tomographic reconstructions were created and the four standard PAO bone cuts-ischial, pubic, anterior, and posterior aspects of the ilium-as well as rotation of the dislocated acetabular bone blocks were simulated for each specimen. Using these data, custom 3D printed bone-drilling templates of the pelvis were manufactured, to guide surgical placement of the PAO bone cuts. An angle fix wedge was designed and printed, to help accurately achieve the predetermined rotation angle of the acetabular bone block. Each specimen underwent a conventional PAO.

Preoperative, post-simulation, and postoperative lateral center-edge angles, acetabular indices, extrusion indices, and femoral head coverage were measured and compared; *P* and *t* values were calculated for the above-mentioned measurements while comparing preoperative and postoperative data, and also in post-simulation and postoperative data comparison. All 10 PAO osteotomies were successfully completed using the 3D printed bone-drilling template and angle fix wedge. No osteotomy entered the hip joint and a single posterior column fracture was observed. Comparison of preoperative and postoperative measurements of the 10 sides showed statistically significant changes, whereas no statistically significant differences between post-simulation and postoperative values were noted, demonstrating the accuracy and utility of the 3D printed templates.

The authors concluded that the application of patient-specific 3D printed bone-drilling and rotation templates in PAO is feasible and may facilitate improved clinical outcomes, using precise presurgical planning and reduced surgical complications with the precisely guided bone drilling.

## CONFLICT OF INTEREST STATEMENT

None declared.
